# Generalised Extreme Value Distributions Provide a Natural Hypothesis for the Shape of Seed Mass Distributions

**DOI:** 10.1371/journal.pone.0121724

**Published:** 2015-04-01

**Authors:** Will Edwards, Angela T. Moles, Caroline Chong

**Affiliations:** 1 College of Marine and Environmental Sciences, James Cook University, Cairns, Australia; 2 Centre for Tropical Biodiversity and Climate Change, James Cook University, Cairns, Australia; 3 Evolution & Ecology Research Centre, School of Biological, Earth and Environmental Sciences, UNSW, Sydney, Australia; 4 Current Address: Research School of Biology, Australian National University, Canberra, Australia; York U, CANADA

## Abstract

Among co-occurring species, values for functionally important plant traits span orders of magnitude, are uni-modal, and generally positively skewed. Such data are usually log-transformed “for normality” but no convincing mechanistic explanation for a log-normal expectation exists. Here we propose a hypothesis for the distribution of seed masses based on generalised extreme value distributions (GEVs), a class of probability distributions used in climatology to characterise the impact of event magnitudes and frequencies; events that impose strong directional selection on biological traits. In tests involving datasets from 34 locations across the globe, GEVs described log10 seed mass distributions as well or better than conventional normalising statistics in 79% of cases, and revealed a systematic tendency for an overabundance of small seed sizes associated with low latitudes. GEVs characterise disturbance events experienced in a location to which individual species’ life histories could respond, providing a natural, biological explanation for trait expression that is lacking from all previous hypotheses attempting to describe trait distributions in multispecies assemblages. We suggest that GEVs could provide a mechanistic explanation for plant trait distributions and potentially link biology and climatology under a single paradigm.

## Introduction

In large comparative datasets, distributions of functionally important plant traits such as seed mass, photosynthetic capability, plant height and leaf size span orders of magnitude [1,2,3], are uni-modal, and heavily skewed [4]. Such data are usually log-transformed “for normality” before analysis. However, we have remarkably little theoretical or empirical evidence to support the idea that traits are log-normally distributed. While normality is improved by log-transformation, few, if any, trait distributions are truly normal on a log scale [4]. Further, it is not clear why we should expect traits to be log-normally distributed. The most recent proposal for the skewed nature of trait distributions invokes the action of a lower bound to the physical dimension of a given trait, while the upper bound is limitless [4], or at least limited by natural selection acting upon trade-offs associated with benefits and costs of increasingly larger trait values [5]. While this theory could explain the right skew of trait data, it does not necessarily lead to the expectation of a log-normal data distribution. In this paper, we propose an alternative hypothesis—that traits follow generalised extreme value distributions (GEVs). We show that in addition to providing a biologically meaningful mechanism for generating the observed trait distributions, GEVs provide a substantially better fit to seed mass distributions across coexisting species than do traditional log-normal distributions, and may give insight into large geographical trends in trait distributions not generated by conventional normalising statistics.

Species occupying the same local environment share one thing: they must each be able to persist in the face of the physical conditions and physiological stresses experienced there. Trait values determine the capacity of species to respond to hazards of different magnitudes through time and thus define the set of environmental conditions each species is capable of enduring [6,7,8]. But, traits themselves are modified by the conditions in which species exist.

Physically and physiologically challenging events influence the evolution of traits by enforcing strong directional selection more so than “average” conditions [9,10], and can also determine the suite of trait values found among co-existing species by acting as a mechanism for species sorting or species filtering [11]. These effects might be expected to result in a match between the general shape of trait value distributions and the shape of the distribution describing the events associated with extremes to which individual species traits respond.

Extreme value distributions (EVDs) describe the relationship between the return frequency and magnitude of environmental variables and thus characterise the regime of physical stresses [e.g. drought, rain, temperature, etc.; 12,13,14] experienced in a given location. Unlike standard normalising statistics, they are a family of distributions that explicitly model tail behaviour (either upper or lower). There are two approaches to examining tail behaviour; either a generalized Pareto distribution is fitted to the upper tail of a large continuous data set, or a generalised extreme value (GEV) distribution is fitted to maximum values identified within given periods (i.e. block maxima). GEVs are described by a density function dependent upon three parameters:

$$F\left(\text{x};\mu ,\sigma ,\xi \right)=exp\left\{-{\left[1+\xi \left(\text{x}-\mu \right)/\sigma \right]}^{-1∕\xi }\right\}$$

with *μ =* location parameter (analogous to the mean in normalising statistics) and *σ* and *ξ* the scale and shape parameters respectively. The range of definition of the GEV distribution depends on the shape parameter *ξ*. The shape parameter (*ξ*) governs the tail behaviour and identifies the distribution as belonging to one of three sub-families of distributions; Type 1 Gumbel (unbounded, *ξ =* 0), Type 2 Fréchet (lower tail bounded, heavy upper tail *ξ* > 0) or Type 3 (negative) Weibull (upper tail bounded and short, *ξ* < 0). Notably, the Gumbel and Fréchet models relate to maxima (large extreme values) whereas the Weibull model relates to minima (small extreme values). Understanding the nature of the shape parameter is especially important, since it describes whether extreme events are likely to occur. Failure to address the possibility of heavy tailed distributions in the face of changing environments can introduce error into climatic and economic trend analyses, as well as in our predictions of the impact of ‘non-standard’ processes, including large climatic events, on biological communities [8,15].

EVDs have been used to model weather and climatic problems where the statistically extreme events are of critical interest and impact, for example temperature maxima and minima, wind, precipitation, and flood discharge [15,16,17,18,19,20]. Since these infrequent and stochastic events are also those that impose “critical response thresholds” [sensu 8] and exert strongest selection pressures on biological processes [12,13,14], they represent a natural hypothesis for the shape of trait distributions among co-occurring species by assuming direct linkage between trait expression and the characteristics of the selective environment in which species exist. There is compelling evidence that biological trait distributions including maximum plant height, specific leaf area (SLA), leaf nitrogen content, and effective dispersal distances deviate from the mean trend of expected distributions or fail to conform to log-normal expectations [4,10]. Here, we test the idea that GEV distributions may better approximate biological trait distributions using a single plant trait; seed mass. Seed mass is an ideal choice, since it is known to be correlated with survival through many of the hazards plants face during establishment, including shade, competition with established plants or other seedlings, burial under soil or litter, nutrient deprivation, drought, and herbivory (reviewed in [1,21]) which combined determine the regeneration niche of individual species [22].

## Materials and Methods

We used seed mass data taken from 30 published and unpublished datasets of co-existing species from locations across the world. Where individual studies reported groups of species that were identified as coming from unique locations, these datasets were divided into subsets representing each sample location (three cases, total number of unique geographic locations = 34, S1 Table). To determine whether GEVs provide a better fit to biological data than do traditional normal distributions, we fitted both distributions to each log_10_ dataset [23] and compared the fit based on information theoretic criteria; i.e. ΔAICc values [24], which accounts for increased explanatory ability expected in a model with greater number of fitted parameters. When ΔAICc values < 2, information lost in fitting competing models is comparable and both can be considered equally likely as possible candidates to explain the observed distribution. When ΔAICc values > 10, the model with the lower AICc value clearly explains some component of the total variation in the observed distribution that the second model does not, and the second model can be considered as having essentially no support [13,24]. ΔAICc values falling between >2 - < 10 show increasingly less support. We report the number of cases where comparisons reveal equal support, substantial support for one or other model, or support for one model only and examine whether normal or GEV models were most informative due to differences in sample size, or geographical position (absolute latitude) via simple t-tests. Further, we calculated the evidence ratio based on Akaike weights to assess the normalised probability of the GEV being the preferred model.

Maximum likelihood methods in extRemes [13] and nsRFA [23] were used to generate parameter estimates for the GEV distributions, as well as a likelihood ratio test to test the shape parameter against a null hypothesis of an underlying Gumbel distribution (i.e. *ξ =* 0). To determine whether GEVs provide additional insight into patterns in the distribution of seed mass across locations that are not revealed under standard normalising distributions, we first examined differences in slope and intercept in the relationships between “mean” seed mass determined from both normal and GEV distributions and latitude by simply comparing slope and intercepts from the two models generated via regression. We then used regression to explore tendencies for change in the shape parameter associated with the GEV fit and latitude, and logistic regression to assess changes in the probability that the distribution would conform to the Gumbel hypothesis in association with latitude.

## Results

We found compelling evidence that the GEV model provides a better explanation of log_10_ seed mass distributions than does the normal distribution. First, based on ΔAIC values alone, information lost due to fitting a GEV distribution was lower than that lost in fitting a normal distribution in 18/34 (55%) cases (S1 Table). There were also nine cases where the normal distribution was identified as the best approximating model, but GEV models returned ΔAICc < 2 (S1 Fig). Together, these represent 27/34 (79%) tests in which the GEV was at least as well supported as the most likely representation of the true distribution as was a normal distribution (S1 Table). Further, the normal distribution should be discarded entirely as an alternative to GEV in 20% of cases (normal ΔAICc > 10), whereas the reverse occurred just once (S1 Table). There was no difference in either absolute latitude (*t* = 0.6313, df = 31.77, P = 0.53) nor sample size (*t* = 1.44, df = 31.87, p = 0.16) between groups where GEV or normal distributions were identified as best approximating models based on ΔAIC. These results were mirrored when model support was determined on the basis of the normalised probability of the GEV (S1 Table). For example, evidence ratios in support of the GEV as the preferred model ranged from 0.001to 0.999, suggesting cases where GEV and other cases where normal were best approximations. Evidence ratios were, however, located strongly in favour of the GEV models. In ten cases the probability of the GEV being the most likely model was greater than 0.9, which decreased to 18 cases where the probability was greater than 0.5. The converse (i.e. where probability of GEV being favoured was less than 0.1) occurred just three times (S1 Table).

**Fig 1:**
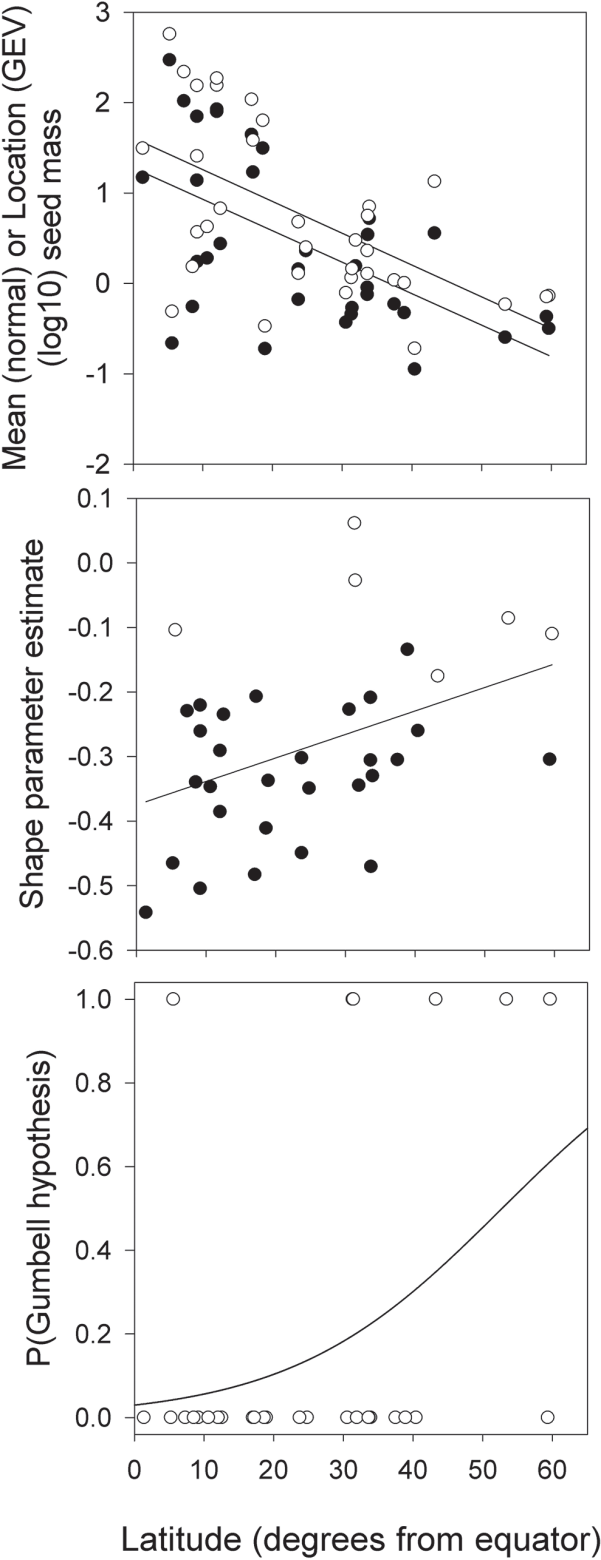
Relationships between parameters describing seed mass distributions and latitude. (A) The equivalent relationships between the mean (estimated under an assumption of a normal distribution; open symbols) and latitude, and the location parameter (estimated under an assumption of GEV model; solid symbols). (B) Increase in magnitude of the GEV shape parameter (*ξ*) with latitude (open symbols show datasets where shape parameter *ξ* not different from zero—see S1 Table). (C) Change in probability that given seed mass distribution will conform to the Gumbel-type with latitude.

Of the 34 datasets, 28 failed the test of a Gumbel-type distribution, and indicate substantial skew (deviation from normality) (S1 Table). In all of these cases the GEV shape parameter was significantly less than zero, that is, small seed sizes are more frequent in these datasets than is estimated by mean species trends (i.e.; a Weibull-type distribution). The contrasting pattern, where large seed size occurs more frequently than expected was never detected. There was no significant difference in slope or intercept between models fitted with location estimates generated from GEV or normal distributions and latitude. Both relationships were significant (*F*_1,32_ = 8.07, P < 0.01 and *F*_1,32_ = 8.17, P <0.01, respectively) and had overlapping estimates (mean ± SE) for slope (normal = -0.0336 ± 0.009; GEV = 0.0334 ± 0.009) and intercept (normal = 1.57 ± 0.25; GEV = 1.25 ± 0.25) (Fig 1A). There was a significant positive relationship between the shape parameter and latitude (*F*_1,32_ = 6.42, P = 0.016, *R* ^2^ = 0.167; Fig 1B), corresponding to a significant increase in the likelihood distributions would change from Weibull (*ξ <* 0) to Gumbel (*ξ =* 0) types in association with increasing distance from the equator (logistic regression P_1,32_ = 0.018), and latitude was able to explain 17.46% of the total deviance (Fig 1C).

## Discussion

Our results show that GEVs fit log_10_ seed mass data as well, and in many cases better than normal distributions. We also show a systematic trend in the shape parameter of seed mass distributions to increase in association with latitude. One possible reason for this is that maximum body size (plant height) is greater at lower latitudes [25]. Larger body size confers greater competitive ability [26], and in plants, larger growth forms also allow for greater investment in seed mass [27]. This results in seed mass distributions closer to the equator including some species with very large seeds. The existence of the few large seeds exerts strong influence on the parameter determinations of the GEV fit, especially the location. This, in turn, results in the identification of a heavy lower tail (an attribute of the shape parameter) in datasets from lower latitudes. In short, closer to the equator small seeds are more frequent than estimated by mean species trends. At higher latitudes there are fewer “comparatively large” seeds; a pattern independent of the absolute magnitude of seed mass values at any single latitude [28].

At present our hypothesis is based on the assumption that plant traits such as seed mass fit GEVs because they are driven by the occurrence of climatic processes such as temperature, wind storm damage and stream flows that themselves follow GEVs [8,14,17,19]. Ultimately, the true test of this idea will require an ability to compare the shape of trait value distributions against the shape of return level plots generated from long-term climatic records from the same location. At present this information is unavailable for the datasets we have used. Nevertheless, as we have demonstrated, the use of GEVs may provide greater insight than the common log normal expectation which has persisted, even in the face of overwhelming evidence that it is unrealistic [4]. We agree that there are good reasons why logarithmic scales are often an appropriate dimensionality on which to examine functional traits [29]. First, log transformation can stabilise variances across the generally large size range that most trait distributions span. Second, within a single species, multiplicative processes such as rates of cell division that ultimately determine structural size, are likely to vary between individuals and between organs within individuals, and this should lead to an expectation of normality after log transformation. This is not true when comparisons are made across species, however. Reproductive, growth form, growth rate and longevity trade-offs comprise the life-history attributes of individual species. The expression of life-history trait combinations in each extant species represents a single successful strategy for persistence in its current environment and are the outcomes of natural selection working on species values in isolation of all other species. In this way, expression of individual traits within an assemblage of co-occurring species is fundamentally different from that of a normally distributed multiplicative process. GEVs, on the other hand, describe the return frequency distribution of events that impose strong directional selection in natural populations and that profoundly influence trait evolution [9,10]. They characterise the full range of disturbance events experienced in a location to which all individual species’ life histories could respond. If true, GEVs may thus provide a natural, biological explanation for trait expression that is lacking from all previous hypotheses attempting to describe trait distributions in multispecies assemblages.

The use of GEVs is well developed in climatology and economic theory, but under-utilised in biology and ecology. If traits reflect GEV distributions as our analyses imply, explanations for broad geographic patterns in trait expression should be sought as a function of species’ responses to differences in return frequencies of critical response thresholds between locations. Some progress in incorporating GEVs in a biological context has been made; for instance, we have recently shown differences in the hydrologic disturbance regime between mainstem and tributary stream types are reflected in population genetic distinctiveness in *Melaleuca leucadendra* (L.), a major vegetation type in dry tropics flood landscapes [30]. GEVs have also been employed in forestry, where fitting bounded GEVs to inventories of large trees within plots has been used to try to predict the numbers of trees in smaller (unrecorded) size classes [31], and in cocoa, where mean seed weight per pod “showed a generalised extreme value distribution” ([32], page 219). Unfortunately, Cilas *et al*. [32] did not provide the parameters describing the distribution of mean seed weight per pod and did not test against log-normality. All above studies focus of single species. Thus we are unable to position these within-species descriptions within the context of our across-species and across-location comparisons of trait distributions drawn from entire assemblages. Nevertheless, an events-based framework based on EVDs might have broad application across a range of ecological disciplines where persistence relies on individual traits (or trait combinations) permitting avoidance of critical failure of biological processes given some level of stress. Historically, the conceptual basis for the importance of event frequencies and magnitudes has been associated with some fields. For example, the study of the impacts of fire events, where both fire intensity and fire frequency define the “fire regime” [33] is an events-based construct. Despite this, we know of no previous studies explicitly assuming a GEV-type distribution for the range of values for traits that species display in fire-prone regions in response to these events. This could be one avenue of possible future research. There is also possible application in the determination of species’ distributional limits, especially in the context of events imposing physiological stresses that represent fatal conditions. For example, critically high leaf temperatures cause localised cavitation and wilting based on traits associated with hydraulic and thermal capacitance and evaporative cooling [34]. Further, there is also no necessary restriction for this approach to apply only to plants. Again, in the context of distributional limits, in north east Queensland, Australia, for example, the distribution of the green ringtail possum (*Pseudochirops archeri*), one of the few large mammals for which detailed physiological information about thermal tolerance is known, is well predicted by the experience of excess dehydration that occurs when extreme temperatures (>30^°^C) persist for more than 6 h per day over 4–6 days or more [35]. The field of hydrology also provides classical examples of extreme event analysis applied to drought and floods that regulate animal populations whereby fish persistence and productivity are governed by the extreme flow variability and "boom and bust" ecology of desert river systems [36].

## Conclusions

The GEV approach represents a new framework that could be used to explore connections between physical and biological processes. We propose that linking plant traits to return levels of climatic-induced stress at a local scale via GEVs has the potential to refine our understanding of all scales of biological organisation and change; from individual, population and community differences, to species geographic tolerances, responses to changing climate and patterns in global net primary productivity. Adopting this novel conceptual approach to examine biological processes could refine current understanding in a range of fundamental ecological and biological outcomes, increase our understanding of the mechanisms allowing a diversity of life-histories to co-exist in single locations and has the potential to unite biology and climatology under a single paradigm. The application of GEVs to biological problems is still in its infancy. We strongly encourage further investigation into the possible application of GEVs in all fields of ecology.

## Supporting Information

S1 Fig Comparison of ecdf fit for normal (solid line) and GEV (dashed line) to log10 seed mass datasets where; (A) GEV superior fit, (B) normal superior fit and (C) neither GEV or normal superior fit. Datasets are from: Jurado, E *et al*. Diaspore weight, dispersal, growth form and perenniality of central Australian plants. *J*. *Ecol*. **79**, 811–830 (1991), Lord, J. *et al*. Larger seeds in tropical floras: Consistent patterns independent of growth form and dispersal mode. *Journal of Biogeography* **24** (1997), Juniper, P. (pers. comm.).
(DOCX)

S1 Table Datasets, decimal latitude and longitude, sample size (number of species), AICc values and the results (and parameter estimates) of normal and GEV distribution model fitting for 34 log_10_-transformed seed mass datasets. Best approximating model based on AICc is indicated by *. Also shown is the result of log-likelihood ratio (Chi-sq) test of the goodness of fit of the GEV to a Gumbel distribution, which tests divergence of the shape parameter from zero, and normalised probability of preferential support for GEV over normal models.
(DOCX)
