# Supplementary material for: Generalised Extreme Value Distributions Provide a Natural Hypothesis for the Shape of Seed Mass Distributions
Source: PLoS One. 2015 Apr 1;10(4):e0121724. doi: 10.1371/journal.pone.0121724 (PMC4382290; doi:10.1371/journal.pone.0121724)
Supplement: S1 Fig — Datasets are from: Jurado, E et al. Diaspore weight, dispersal, growth form and perenniality of central Australian plants. J. Ecol. 79, 811–830 (1991), Lord, J. et al. Larger seeds in tropical floras: Consistent patterns independent of growth form and dispersal mode. Journal of Biogeography 24 (1997), Juniper, P. (pers. comm.). (DOCX) [file pone.0121724.s001.docx]

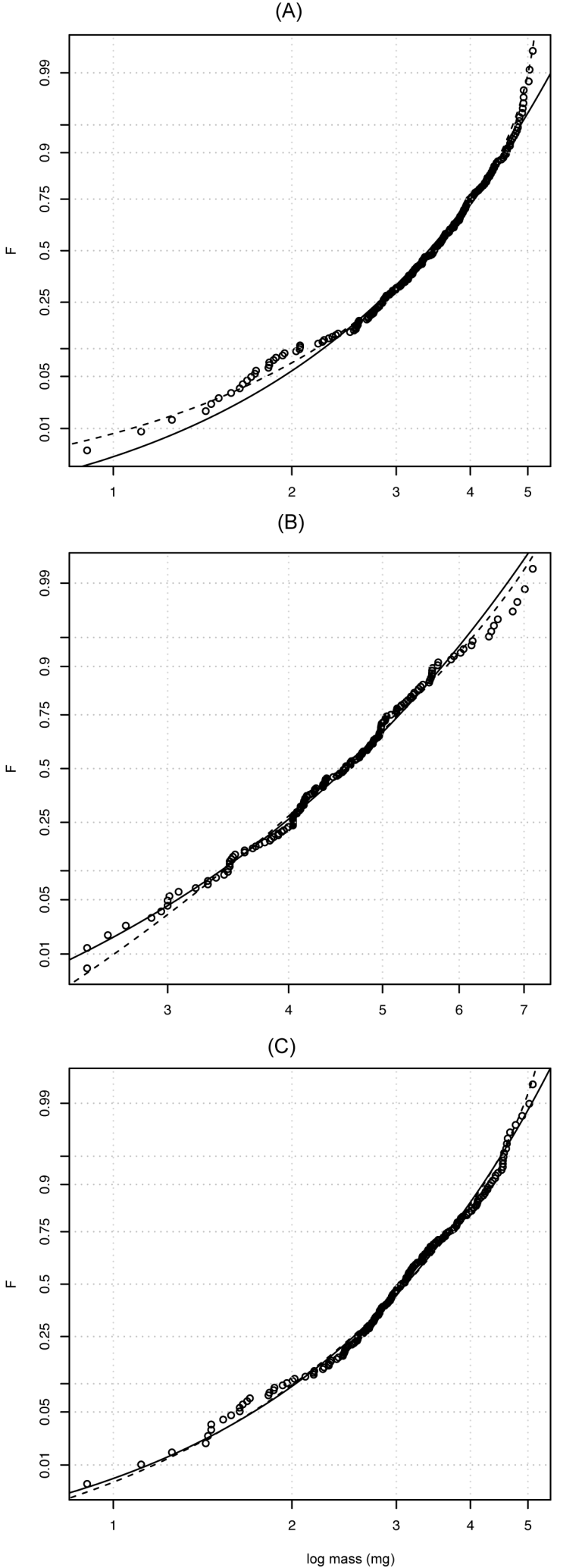


Figure A in S1 Figure. Comparison of ecdf fit for normal (solid line) and GEV (dashed line) to log10 seed mass datasets where; (A) GEV superior fit, (B) normal superior fit and (C) neither GEV or normal superior fit. Datasets are from: (A) Jurado, E., Westoby, M. & Nelson, D. Diaspore weight, dispersal, growth form and perenniality of central Australian plants. *J. Ecol.* **79**, 811-830 (1991), (B) Lord, J. *et al.* Larger seeds in tropical floras: Consistent patterns independent of growth form and dispersal mode. *Journal of Biogeography* **24** (1997), (C) Juniper, P. (pers. comm.).
