# Supplementary material for: Generalised Extreme Value Distributions Provide a Natural Hypothesis for the Shape of Seed Mass Distributions
Source: PLoS One. 2015 Apr 1;10(4):e0121724. doi: 10.1371/journal.pone.0121724 (PMC4382290; doi:10.1371/journal.pone.0121724)
Supplement: S1 Table — Best approximating model based on AICc is indicated by *. Also shown is the result of log-likelihood ratio (Chi-sq) test of the goodness of fit of the GEV to a Gumbel distribution, which tests divergence of the shape parameter from zero, and normalised probability of preferential support for GEV over normal models. (DOCX) [file pone.0121724.s002.docx]

Table A in S1 Table. Datasets, decimal latitude and longitude, sample size (number of species), AICc values and the results (and parameter estimates) of normal and GEV distribution model fitting for 34 log_10_-transformed seed mass datasets. Best approximating model based on AICc is indicated by *. Also shown is the result of log-likelihood ratio (Chi-sq) test of the goodness of fit of the GEV to a Gumbel distribution, which tests divergence of the shape parameter from zero, and normalised probability of preferential support for GEV over normal models.

| Dataset | Latitude | Longitude | n | AICc (NORM) | AICc (GEV) | mean (NORM) | variance (NORM) | location (GEV) | scale (GEV) | shape (GEV) | Test  H_o_ *ξ =* 0 | Normailsed probability of GEV |
| --- | --- | --- | --- | --- | --- | --- | --- | --- | --- | --- | --- | --- |
| ^1^ | 9.15 | -79.85 | 147 | 451.2 | 434.5* | 1.41 | 1.11 | 1.14 | 1.20 | -0.50 | <0.001 | 0.999 |
| ^2^ | -33.88 | 151.21 | 40 | 39.8* | 40.9 | 0.87 | 0.38 | 0.73 | 0.37 | -0.26 | 0.031 | 0.365 |
| ^3^ | 9.17 | -79.85 | 69 | 195.2* | 197.3 | 0.59 | 0.97 | 0.24 | 0.96 | -0.26 | 0.005 | 0.259 |
| ^4^ | -31.30 | 17.9 | 37 | 98.01 | 92.21* | 0.06 | 0.86 | -0.34 | 0.63 | 0.06 | 0.66 | 0.948 |
| ^5^ | 59.65 | 17.95 | 196 | 496.6 | 486* | -0.14 | 0.86 | -0.50 | 0.75 | -0.11 | 0.055 | 0.955 |
| ^6^ | -12.00 | -72 | 204 | 560.4 | 559.8* | 2.19 | 0.95 | 1.90 | 1.04 | -0.39 | <0.001 | 0.574 |
| ^7^ | 18.92 | -96.91 | 68 | 160.5* | 161.2 | -0.47 | 0.76 | -0.72 | 0.78 | -0.34 | 0.001 | 0.413 |
| ^8^ | 53.38 | -1.46 | 73 | 188.5 | 184.5* | -0.23 | 0.86 | -0.60 | 0.74 | -0.09 | 0.26 | 0.88 |
| ^9^ | -17.00 | 145.5 | 203 | 751.8 | 734.7* | 2.03 | 1.53 | 1.65 | 1.64 | -0.48 | <0.001 | 0.999 |
| ^10^ | 5.22 | -58.8 | 307 | 927.4 | 903.1* | 2.76 | 1.09 | 2.47 | 1.18 | -0.47 | <0.001 | 0.999 |
| ^11^ | -33.67 | 151.33 | 48 | 117.1 | 115.4* | 0.75 | 0.80 | 0.54 | 0.87 | -0.47 | <0.001 | 0.701 |
| ^12^ | 18.60 | -95.11 | 125 | 370 | 365.5* | 1.80 | 1.05 | 1.49 | 1.11 | -0.41 | <0.001 | 0.905 |
| ^13^ | -17.18 | 145.63 | 163 | 439.6* | 440.3 | 1.58 | 0.92 | 1.23 | 0.88 | -0.21 | <0.001 | 0.413 |
| ^14^ | -23.70 | 133.86 | 222 | 582.3 | 571.5* | 0.40 | 0.89 | 0.16 | 0.95 | -0.45 | <0.001 | 0.996 |
| ^15^ | 24.78 | -99.53 | 110 | 313.7 | 313* | 0.67 | 0.99 | 0.36 | 1.01 | -0.35 | <0.001 | 0.587 |
| ^16^ | 9.15 | -79.85 | 48 | 130.7* | 132.5 | 2.19 | 0.90 | 1.85 | 0.87 | -0.22 | 0.03 | 0.289 |
| ^17^ | -31.42 | -66.4 | 85 | 236.7 | 229.4* | 0.16 | 0.95 | -0.27 | 0.77 | -0.03 | 0.79 | 0.975 |
| ^18^ | -12.50 | 132.5 | 243 | 617.3* | 623.8 | -0.11 | 0.86 | -0.43 | 0.86 | -0.23 | <0.001 | 0.037 |
| ^18^ | -23.70 | 133.86 | 197 | 493.7 | 493.6* | 0.11 | 0.84 | -0.18 | 0.85 | -0.30 | <0.001 | 0.512 |
| ^18^ | -30.52 | 145.13 | 230 | 674.9* | 684.7 | 0.83 | 1.04 | 0.44 | 1.05 | -0.23 | <0.001 | 0.007 |
| ^19^ | -7.27 | 36.35 | 123 | 315.6* | 319.3 | 2.34 | 0.86 | 2.02 | 0.85 | -0.23 | <0.001 | 0.136 |
| ^20^ | 8.52 | -83.41 | 258 | 892.3 | 890.5* | 0.19 | 1.35 | -0.26 | 1.40 | -0.34 | <0.001 | 0.711 |
| ^21^ | 1.35 | 103.78 | 151 | 544.4 | 526.1* | 1.49 | 1.45 | 1.17 | 1.58 | -0.54 | <0.001 | 0.999 |
| ^22^ | 59.32 | 18.05 | 53 | 107.9* | 109.5 | -0.15 | 0.64 | -0.37 | 0.65 | -0.30 | 0.005 | 0.310 |
| ^23^ | -33.60 | 151.23 | 50 | 120.6* | 121.9 | 0.04 | 0.77 | -0.27 | 0.77 | -0.31 | 0.014 | 0.343 |
| ^23^ | -37.45 | 148.25 | 80 | 149.8* | 151.8 | 0.11 | 0.60 | -0.12 | 0.58 | -0.21 | 0.003 | 0.269 |
| ^24^ | 12.00 | 76 | 97 | 276.6 | 276.3* | 2.27 | 0.99 | 1.93 | 0.97 | -0.29 | 0.002 | 0.537 |
| ^25^ | 40.40 | -3.68 | 70 | 138.2* | 139.8 | -0.72 | 0.63 | -0.95 | 0.62 | -0.26 | 0.006 | 0.310 |
| ^26^ | 10.60 | -67.03 | 33 | 86.29 | 86.19* | -0.31 | 0.84 | -0.66 | 0.73 | -0.10 | 0.33 | 0.512 |
| ^26^ | 5.58 | -66.88 | 148 | 443.8* | 448 | 0.63 | 1.07 | 0.28 | 1.15 | -0.35 | <0.001 | 0.109 |
| ^27^ | 43.25 | 141.83 | 50 | 181.1* | 181.3 | 1.13 | 1.42 | 0.56 | 1.29 | -0.18 | 0.36 | 0.475 |
| ^28^ | 38.90 | 46.83 | 297 | 720.4 | 710.9* | 0.00 | 0.81 | -0.32 | 0.73 | -0.13 | <0.001 | 0.991 |
| ^29^ | -31.92 | 115.82 | 79 | 205.1* | 207.7 | 0.48 | 0.86 | 0.19 | 0.93 | -0.34 | <0.001 | 0.214 |
| ^30^ | -33.60 | 151.23 | 304 | 954.1* | 967.8 | 0.36 | 1.15 | -0.04 | 1.25 | -0.31 | <0.001 | 0.001 |

Datasets

1. Augspurger, C. K. Mass allocation moisture content and dispersal capacity of wind-dispersed tropical diaspores. *New Phytol.* **108**, 357-368 (1988).

2. Auld, T. D. & O’Connell, M. A. Predicting patterns of post-fire germination in 35 eastern Australian Fabaceae. *Australian Journal of Ecology* **16**, 53-70 (1991).

3. Dalling, J. W., Hubbell, S. P. & Silvera, K. Seed dispersal, seedling establishment and gap partitioning among tropical pioneer trees. *J. Ecol.* **86**, 674-689 (1998).

4. De Villiers, A. J., Van Rooyen, M. W. & Theron, G. K. Seed bank classification of the Strandveld Succulent Karoo, South Africa. *Seed Science Research* **12**, 57-67 (2002).

5. Eriksson, A. & Eriksson, O. Seedling recruitment in semi-natural pastures: the effects of disturbance, seed size, phenology and seed bank. *Nordic Journal of Botany* **17**, 469-482 (1997).

6. Foster, S. A. & Janson, C. H. The relationship between seed size and establishment conditions in tropical woody plants. *Ecology* **66**, 773-780 (1985).

7. Funes, G., Basconcelo, S., Diaz, S. & Cabido, M. Seed size and shape are good predictors of seed persistence in soil in temperate mountain grasslands of Argentina. *Seed Science Research* **9**, 341-345 (1999).

8. Grime, J. P. *et al.* A comparative study of germination characteristics in a local flora. *J. Ecol.* **69**, 1017-1059 (1981).

9. Grubb, P. J., Metcalfe, D. J., Grubb, E. A. A. & Jones, G. D. Nitrogen-richness and protection of seeds in Australian tropical rainforest: a test of plant defence theory. *Oikos* **82**, 467-482 (1998).

10. Hammond, D. S. & Brown, V. K. Seed size of woody plants in relation to disturbance, dispersal, soil type in wet neotropical forests. *Ecology* **76**, 2544-2561 (1995).

11. Henery, M. & Westoby, M. Seed mass and seed nutrient content as predictors of seed output variation between species. *Oikos* **92**, 479-490 (2001).

12. Ibarra-Manríquez, G. & Oyama, K. Ecological correlates of reproductive traits of Mexican rain forest trees. *American Journal of Botany* **79**, 383-394 (1992).

13. Juniper, P. (pers. comm.).

14. Jurado, E., Westoby, M. & Nelson, D. Diaspore weight, dispersal, growth form and perenniality of central Australian plants. *J. Ecol.* **79**, 811-830 (1991).

15. Jurado, E., Estrada, E. & Moles, A. T. Characterizing plant attributes with particular emphasis on seeds in Tamaulipan thornscrub in semi-arid Mexico. *Journal of Arid Environments* **48**, 309-321 (2001).

16. Kitajima, K. *The importance of cotyledon functional morphology and patterns of seed reserve utilization for the ecology of neotropical tree seedlings*, University of Illinois, (1992).

17. Diaz, S. (pers. comm.).

18. Lord, J. *et al.* Larger seeds in tropical floras: Consistent patterns independent of growth form and dispersal mode. *Journal of Biogeography* **24** (1997).

19. Msanga, H. P. *Seed Germination of Indigenous Trees in Tanzania, Including Notes on Seed Processing, Storage and Plant Uses*. (Canadian Forest Service, 1998).

20. Mayfield, M. (pers. comm.).

21. Metcalfe, D. J. & Grubb, P. J. Seed mass and light requirements for regeneration in southeast Asian rain forest. *Canadian Journal of Botany* **73**, 817-826 (1995).

22. Milberg, P., Andersson, L. & Thompson, K. Large-seeded species are less dependent on light for germination than small-seeded ones. *Seed Science Research* **10**, 99-104 (2000).

23. Moles, A. T., Warton, D. I. & Westoby, M. Do small-seeded species have higher survival through seed predation than large-seeded species? *Ecology* **84**, 3148-3161 (2003).

24. Murali, K. S. Patterns of seed size, germination and seed viability of tropical tree species in southern india. *Biotropica* **29**, 271-279 (1997).

25. Peco, B., Traba, J., Levassor, C., Sanchez, A. M. & Azcarate, F. M. Seed size, shape and persistence in dry Mediterranean grass and scrublands. *Seed Science Research* **13**, 87-95 (2003).

26. Ramirez, N. Produccion y costo de frutos y semillas entre formas de vida. *Biotropica* **25**, 46-60 (1993).

27. Seiwa, K. & Kikuzawa, K. Phenology of tree seedlings in relation to seed size. *Canadian Journal of Botany* **69**, 532-538 (1991).

28. Thompson, K. *et al.* Seed size, shape and persistence in the soil in an Iranian flora. *Seed Science Research* **11**, 345-355 (2001).

29. Touchell, D. H. & Dixon, K. W. Cryopreservation of seed of Western Australian native species. *Biodivers. Conserv.* **2**, 594-602 (1993).

30. Westoby, M., Rice, B. & Howell, J. Seed size and plant growth form as factors in dispersal spectra. *Ecology* **71**, 1307-1315 (1990).
